# Decreased serum carbohydrate antigen 19–9 levels after neoadjuvant therapy predict a better prognosis for patients with pancreatic adenocarcinoma: a multicenter case-control study of 240 patients

**DOI:** 10.1186/s12885-019-5460-4

**Published:** 2019-03-21

**Authors:** Shuichi Aoki, Fuyuhiko Motoi, Yoshiaki Murakami, Masayuki Sho, Sohei Satoi, Goro Honda, Kenichiro Uemura, Ken-ichi Okada, Ippei Matsumoto, Minako Nagai, Hiroaki Yanagimoto, Masanao Kurata, Takumi Fukumoto, Masamichi Mizuma, Hiroki Yamaue, Michiaki Unno, for the Multicenter Study Group of Pancreatobiliary Surgery (MSG-PBS)

**Affiliations:** 1 Department of Surgery, Tohoku University Graduate School of Medicine, 1-1, Seiryo-machi, Aobaku, Sendai, Miyagi 980-8574 Japan; 2 Department of Surgery, Institute of Biomedical and Health Sciences, Hiroshima University, Hiroshima, 734-8553 Japan; 3 Department of Surgery, Nara Medical University, Nara, 634-8521 Japan; 4 Department of Surgery, Kansai Medical University, Osaka, 573-1010 Japan; 5 Department of Surgery, Tokyo Metropolitan Cancer and Infectious diseases Center Komagome Hospital, Tokyo, 113-8677 Japan; 6 Department of Gastointestinal and Hepato-Biliary-Pancreatic Surgery, Faculty of Medicine, University of Tsukuba, Tsukuba, 305-8575 Japan; 7 Second Department of Surgery, Wakayama Medical University, Wakayama, 641-8510 Japan; 8 Department of Surgery, Kindai University Faculty of Medicine, Osaka, 577-8502 Japan; 9 Department of Surgery, Kobe University Graduate School of Medicine, Kobe, 650-0017 Japan

**Keywords:** Carbohydrate antigen 19–9, Neoadjuvant therapy, Pancreatic cancer

## Abstract

**Background:**

Carbohydrate antigen (CA) 19–9 levels after resection are considered to predict prognosis; however, the significance of decreased CA19–9 levels after neoadjuvant therapy has not been clarified. This study aimed to define the prognostic significance of decreased CA19–9 levels after neoadjuvant therapy in patients with pancreatic adenocarcinoma.

**Methods:**

Between 2001 and 2012, 240 consecutive patients received neoadjuvant therapy and subsequent resection at seven high-volume institutions in Japan. These patients were divided into three groups: Normal group (no elevation [≤37 U/ml] before and after neoadjuvant therapy), Responder group (elevated levels [> 37 U/ml] before neoadjuvant therapy but decreased levels [≤37 U/ml] afterwards), and Non-responder group (elevated levels [> 37 U/ml] after neoadjuvant therapy). Analyses of overall survival and recurrence patterns were performed. Uni- and multivariate analyses were performed to clarify the clinicopathological factors influencing overall survival. The initial metastasis sites were also evaluated in these groups.

**Results:**

The Responder group received a better prognosis than the Non-responder group (3-year overall survival: 50.6 and 41.6%, respectively, *P* = 0.026), but the prognosis was comparable to the Normal group (3-year overall survival: 54.2%, *P* = 0.934). According to the analysis of the receiver operating characteristic curve, the CA19–9 cut-off level defined as no elevation after neoadjuvant therapy was ≤103 U/ml. The multivariate analysis revealed that a CA19–9 level ≤ 103 U/ml, (*P* = 0.010, hazard ratio: 1.711; 95% confidence interval: 1.133–2.639), tumor size ≤27 mm (*P* = 0.040, 1.517; (1.018–2.278)), a lack of lymph node metastasis (*P* = 0.002, 1.905; (1.276–2.875)), and R0 status (*P* = 0.045, 1.659; 1.012–2.627) were significant predictors of overall survival. Moreover, the Responder group showed a lower risk of hepatic recurrence (18%) compared to the Non-responder group (31%), though no significant difference in loco-regional, peritoneal or other distant recurrence were observed between groups (*P* = 0.058, *P* = 0.700 and *P* = 0.350, respectively).

**Conclusions:**

Decreased CA19–9 levels after neoadjuvant therapy predicts a better prognosis, with low incidence of hepatic recurrence after surgery.

**Electronic supplementary material:**

The online version of this article (10.1186/s12885-019-5460-4) contains supplementary material, which is available to authorized users.

## Background

Surgery remains the only curative treatment for pancreatic adenocarcinoma; however, a significant number of patients develop local recurrence or distant metastases after surgical resection. Even in seemingly localized tumors, a high probability exists that tumor cells will spread to distant sites, thus negatively affecting long-term survival [1]. Therefore, surgery alone is not considered sufficient for favorable survival outcomes, with a 5-year overall survival (OS) rate of 8–10% [2, 3], and surgical resection combined with adjuvant chemotherapy results in a modest improvement in survival, with a 5-year OS rate of 21–24% [4–8].

Neoadjuvant therapy is considered a potential approach to improve long-term survival, and several studies involving single institutions and national cohorts, as well as data from prospective phase II trials, have revealed a survival benefit following the administration of neoadjuvant therapy [9–15]. The presumable benefits of neoadjuvant therapy include a decrease in tumor burden, which may result in a higher rate of R0 resection, and facilitation of early treatment for micrometastatic disease, which may be undetectable on preoperative radiological images, but may be then detected in early postoperative stages [9–16]. The identification of surrogate markers that predict prognostic significance in the resection after neoadjuvant therapy would be beneficial to discriminate patients who experience an immediate early recurrence after surgery.

According to several studies, the perioperative carbohydrate antigen (CA) 19–9 level is a useful prognostic indicator for patients who do not undergo preoperative therapy [17–25]. Elevated postoperative CA19–9 levels may reflect the presence of residual cancer cells after surgery, and tumor marker normalization after surgery was shown to be a strong postoperative predictor of clinical outcomes. Therefore, researchers have hypothesized that a decrease in CA19–9 levels (≤37 U/ml) after neoadjuvant therapy also predicts improved survival of patients who undergo neoadjuvant therapy followed by resection. The aim of the present study was to define the prognostic impact of normal CA19–9 levels after neoadjuvant therapy in terms of survival and recurrence patterns.

## Methods

### Study design and data collection

This study retrospectively analyzed patients who underwent R0/1 (macroscopic curative) resection for pancreatic cancer at seven high-volume medical institutions in Japan (Multicenter Study Group of Pancreatobiliary Surgery) between January 2001 and January 2012 (Additional file 1: Table S1). Consecutive patients with pancreatic cancer were registered in prospectively collected databases at each institution, which were then combined into a single common database. This study was approved by the institutional review board of Hiroshima University (No. EKI-747).

### Patient selection

**Fig. 1:**
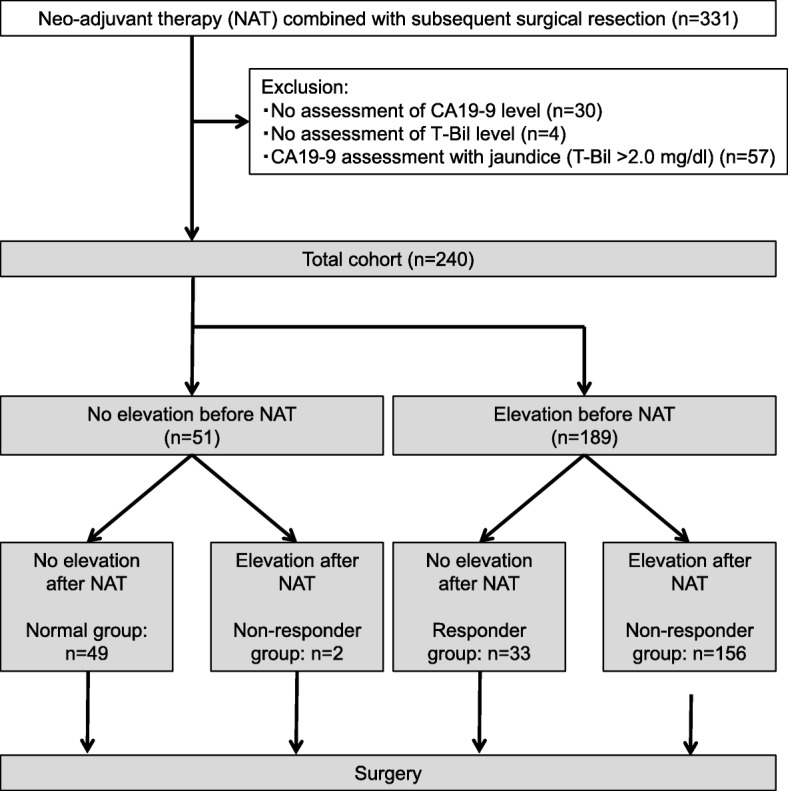
Flowchart of the patient selection process. Among the 331 patients who received neoadjuvant therapy and subsequent resection, patients with no assessment of carbohydrate antigen 19–9 (CA19–9) levels before or after surgery or with a total bilirubin level > 2.0 mg/dL were excluded. The remaining 240 patients were eligible and divided into three groups, the Normal group (sustained decreased CA19–9 levels ≤37 U/ml before and after neoadjuvant therapy), the Responder group (initial high levels that decreased to ≤37 U/ml after neoadjuvant therapy, and the Non-responder group (elevated level > 37 U/ml after neoadjuvant therapy)

The large-scale database included 1401 consecutive patients with invasive ductal adenocarcinoma of the pancreas who underwent R0/1 resection. Patients with tumors other than invasive ductal carcinoma, such as endocrine carcinoma, acinar cell carcinoma, or intraductal papillary mucinous carcinoma were excluded. Among the 1401 patients, 331 were treated with neoadjuvant therapy; the remaining 1070 patients who underwent surgical resection without any preoperative therapy were excluded. Patients with the following characteristics were also excluded (Fig. 1): (1) no assessment of CA19–9 levels before or after surgery; (2) no assessment of total bilirubin levels before neoadjuvant therapy; and (3) a total bilirubin level > 2.0 mg/dL at the time of the CA19–9 assessment (because serum CA19–9 levels may be influenced by biliary obstruction [23]). Finally, 240 patients were eligible for this study. All 240 patients received neoadjuvant therapy before successful R0/1 surgical resection.

For the purposes of this present study, the 240 patients were divided into 3 groups based on CA19–9 alterations observed during the preoperative period. The Normal group included patients without elevated CA19–9 levels (≤37 U/ml) before and after neoadjuvant therapy (*n* = 49, 20%). The Responder group included patients with elevated levels before neoadjuvant therapy and decreased levels (to ≤37 U/ml) after neoadjuvant therapy (*n* = 33, 14%). The Non-responder group included patients with elevated levels before and after neoadjuvant therapy (*n* = 156, 65%) or patients without elevated levels before neoadjuvant therapy who subsequently presented elevated levels after neoadjuvant therapy (*n* = 2, 1%).

### Measurement of serum CA19–9 levels

All serum CA19–9 levels were determined using radioimmunoassay kits at the respective institutions. The manufacturer’s recommended normal upper limit was 37 U/ml. The patients with CA19–9 levels < 2 U/ml were judged to be nonsecretors of CA19–9 due to a lack of the Lewis antigen glycosyl transferase, therefore, they were excluded from this study. When patients with pancreatic head tumors developed obstructive jaundice due to invasion of the biliary tract, biliary drainage was routinely performed. Pre-neoadjuvant therapy CA19–9 levels were measured within a month before the initiation of neoadjuvant therapy. Preoperative levels were measured within a month after the administration of the last dose, while postoperative levels were defined as the lowest levels recorded within a month after surgery.

### Preoperative management

Resectability of pancreatic cancer was evaluated before the initiation of neoadjuvant therapy based on radiographic imaging studies, including thin-sliced abdominal computed tomography or magnetic resonance imaging, as defined by the National Comprehensive Cancer Network guideline. Tumors without any abutment of major vessels, including the superior mesenteric artery, hepatic artery, and the celiac artery, were considered resectable, regardless of the presence of the abutment of the portal vein/superior mesenteric vein. Tumors with reconstructible impingement of the portal vein/superior mesenteric vein and/or major arterial abutment within 180° (assumed to be separable at surgery) were considered borderline resectable. Tumors with irreparable involvement of the portal vein/superior mesenteric vein or major arterial abutment exceeding 180° were considered to be unresectable. The presence of distant metastases (M1) and peritoneal dissemination was considered contraindications for resection.

The type of preoperative therapy and the treatment period depend on the standard protocol followed at each institution (Additional file 1: Table S2); treatment regimens pertaining to neoadjuvant therapy were predetermined at each institution, regardless of tumor progression or treatment efficacy. Approximately 2–4 courses of systemic chemotherapy with gemcitabine (*n* = 23, 10%), gemcitabine plus S-1 (*n* = 91, 38%), or other regimens (*n* = 1, 0%) were administered. Targeted chemoradiotherapy for specific fields, including the primary pancreatic tumor, the surrounding arteries, and the retroperitoneal soft tissue, was performed. The total radiation dose was 45–50.4 Gy, and systemic chemotherapy with gemcitabine (*n* = 64, 27%), S-1 (*n* = 49, 20%), gemcitabine plus S-1 (*n* = 10, 4%), or 5-fluorouracil plus cisplatin (*n* = 2, 1%) was part of the regimen. Radiological responses were evaluated using Response Evaluation Criteria in Solid Tumors (RECIST) a few weeks after the administration of the last dose.

### Surgical procedure and postoperative treatment

Patients underwent pancreaticoduodenectomy with or without pylorus ring resection, distal pancreatectomy with or without celiac axis resection, or total pancreatectomy with extended lymph node dissections. In this study, all surgical procedures were divided into two categories: distal pancreatectomy or pancreaticoduodenectomy. Distal pancreatectomy with celiac axis resection (*n* = 26) was classified as distal pancreatectomy, and total pancreatectomy (*n* = 11) was classified as pancreaticoduodenectomy. Partial or circular resection of the portal vein/superior mesenteric vein was performed if the tumor was not separable from the portal vein/superior mesenteric vein. Regarding the residual tumor status, surgery with microscopically negative margins was defined as a R0 resection, and surgery with microscopically positive margins was defined as a R1 resection. All patients with R2 resection (macroscopic residual tumor) were excluded from this study.

Because the efficacy of postoperative gemcitabine-based adjuvant chemotherapy has been previously reported [4–6], adjuvant gemcitabine therapy was administered routinely during the analyzed period at all institutions. When local recurrence or distant metastasis was present, further treatments were not specified, and various chemotherapies and/or radiation therapies were administered according to the clinical indications.

### Statistical analysis

The Ҳ^2^ test was used to compare categorical variables. The Mann-Whitney U-test was used to evaluate continuous variables and data from more than two groups were analyzed using one-way ANOVA. The Kaplan-Meier method was used to generate survival curves based on preoperative alterations in CA19–9 levels. Statistical analyses of the survival curves were performed with the Cox proportional hazard model and hazard ratio (HR) and 95% CI were calculated. The Cox proportional hazards model was applied by including categorized versions of variables that were significant in the initial log-rank tests in a forward stepwise regression analysis. *P* values < 0.05 were considered statistically significant. JMP software (SAS Institute, Cary, NC) was used for all statistical analyses.

## Results

### Demographic data and pathological assessment

This study included 119 male and 121 female patients with a median age of 67 years (Table 1). The post-treatment tumor size of the Normal group was significantly smaller than the Responder and Non-responder groups (*P* = 0.026). After completing neoadjuvant therapy (median 69 days), no differences in the number of patients with a complete response, partial response, stable disease, or progressive disease were observed between groups (*P* = 0.096). According to the Union for the International Cancer Control classification, regional lymph node metastasis was more frequently observed in the Non-responder group (59%) than in the Normal or Responder groups (49 and 36%, respectively) (*P* = 0.042).

**Table 1:** Descriptive statistics for total cohort and two subgroups stratified by preoperative CA19–9 alteration

	Total cohort	Subgroups			*P* value
		Normal	Responder	Non-responder	
Number	240	49	33	158	
Gender (*n*)					
Male:Female	119:121	23:26	16:17	80:78	0.895
Age (year)					
Median (range)	67 (36–83)	65 (38–79)	68 (36–78)	66 (41–83)	0.881
Pre-neoadjuvant therapy CA19–9 value (U/ml)					
Median (range)	152.7 (2.0–87,100.0)	12.9 (2.0–36.4)	84.0 (39.5–1129.0)	344.8 (32.0–87,100.0)	0.151
Post-neoadjuvant therapy CA19–9 value (U/ml)					
Median (range)	57.7 (2.0–37,370.0)	8.5 (2.0–30.0)	21.0 (8.1–33.0)	134.5 (38.0–37,370.0)	0.148
% decrease of CA19–9 value (%)					
Median (range)	53.2 (−100.0–326.4)	14.7 (− 100.0–100.0)	75.6 (24.9–90.2)	54.1 (− 326.4–96.4)	<.0001
Post-surgical CA19–9 value (U/ml)					
Median (range)	18.6 (2.0–3929.4)	7.0 (2.0–35.0)	9.3 (4.0–171.0)	35.1 (3.2–3929.4)	0.026
CA19–9 normalization after surgery, n(%)					
yes	164 (68)	49 (100)	29 (88)	86 (54)	
no	76 (32)	0 (0)	4 (12)	72 (46)	<.0001
Post-surgical tumor size (mm)					
Median (range)	27.0 (8.0–70.0)	24.5 (8.0–48.0)	28.0 (10.0–50.0)	28.0 (10.0–70.0)	0.026
Preoperative drainage, *n* (%)					
yes	152 (63)	35 (71)	17 (52)	100 (63)	
no	88 (37)	14 (29)	16 (48)	58 (37)	0.187
NCCN resectability, *n* (%)					
Resectable	132 (55)	32 (65)	21 (64)	79 (50)	
Borderline	108 (45)	17 (35)	12 (36)	79 (50)	0.096
Neoadjuvant therapy, *n* (%)					
Systematic chemotherapy	115 (48)	27 (55)	11 (33)	77 (49)	
Chemoradiotherapy	125 (52)	22 (45)	22 (67)	81 (51)	0.145
Duration of neoadjuvant therapy (day)					
Median (range)	69 (13–120)	69 (22–117)	71 (44–112)	68 (13–120)	0.569
RECIST, *n* (%)					
Complete response	11 (5)	4 (8)	2 (6)	5 (3)	
Partial response,	33 (14)	11 (23)	7 (21)	15 (10)	
Stable disease	192 (80)	33 (67)	24 (73)	135 (85)	
Progressive disease	4 (1)	1 (2)	0 (0)	3 (2)	0.096
Operation, *n* (%)					
Pancreaticoduodenectomy	160 (67)	33 (67)	26 (79)	101 (64)	
Distal pancreatectomy	80 (33)	16 (33)	7 (21)	57 (36)	0.256
N classification (UICC), *n* (%)					
1	130 (54)	24 (49)	12 (36)	93 (59)	
0	110 (46)	25 (51)	21 (64)	64 (41)	0.042
Residual tumor, *n* (%)					
R0	205 (85)	44 (90)	32 (97)	129 (82)	
R1	35 (15)	5 (10)	1 (3)	29 (18)	0.048
Adjuvant therapy, *n* (%)					
yes	205 (85)	42 (86)	27 (82)	136 (86)	
no	35 (15)	7 (14)	6 (18)	22 (14)	0.826
Completion of adjuvant therapy, *n* (%)					
yes	139 (67)	34 (83)	23 (85)	82 (59)	
no	68 (33)	7 (17)	4 (15)	57 (41)	0.002

NCCN National Comprehensive Cancer Network, RECIST response evaluation criteria in solid tumours

Significant difference in pre- and post-neoadjuvant therapy CA19–9 levels were not observed between the Normal, Responder, and the Non-responder groups (*P* = 0.151 and 0.148, respectively). However, significantly lower postsurgical CA19–9 levels were observed in the Responder group than in the Non-responder group (*P* = 0.026). The percent decrease in CA19–9 levels during neoadjuvant therapy was significantly larger and CA19–9 levels after surgery were more frequently normalized in the Responder group (*P* < 0.0001 and *P* < 0.0001, respectively). Difference were not observed between Responder and Non-responder groups stratified according to the type of neoadjuvant therapy (systematic chemotherapy or chemoradiotherapy (*P* = 0.145). Although no difference in the administration of adjuvant therapy was noted (*P* = 0.826), a greater number of patients in the Responder group had completed the initially-planned treatment than patients in the Non-responder group (*P* = 0.002).

A significantly greater number of patients with an R0 status was observed in the Normal and Responder groups (90 and 97%) than in the Non-responder group (82%) (*P* = 0.048). The R1 status was observed in 15% of the entire cohort. In 40% of patients with R1 tumors, chemoradiotherapy was administered as neoadjuvant therapy; combined portal vein resection was performed in 60% of these patients. A significant correlation between R status and the type of neoadjuvant therapy or portal vein resection was not observed (*P* = 0.122 and *P* = 0.150, respectively; not shown).

A significant difference in the resectability by National Comprehensive Cancer Network guideline was not observed between the Normal, Responder and the Non-responder groups (*P* = 0.096). Borderline resectable pancreatic cancer was diagnosed before neoadjuvant therapy in 45% of the cohort. Chemoradiotherapy was performed in 46% of patients with borderline resectable pancreatic cancer, and combined portal vein resection was performed in 71% of these patients. No correlation was observed between the resectability and the type of neoadjuvant therapy (*P* = 0.104); however, combined portal vein resection was more frequently performed in patients with borderline resectable pancreatic cancer than in patients with resectable pancreatic cancer (*P* < 0.0001; not shown).

### Survival analysis stratified by alterations in perioperative CA19–9 levels

**Fig. 2:**
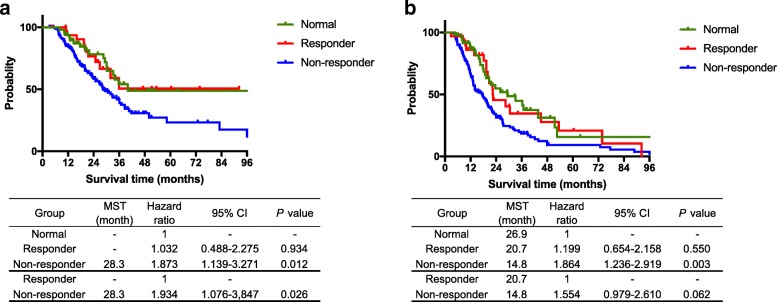
Kaplan-Meier survival curves for three groups. **a** Overall survival (OS) and **b** recurrence-free survival. Patients in the Responder group had a more favorable prognosis than patients in the Non-responder group (3-year OS and recurrence-free survival: 50.6 and 39.9%, and 41.6 and 20.2%, respectively, *P* = 0.026 and *P* = 0.0621, respectively). The prognosis of the Responder group was comparable to the Normal group, with 3-year OS and recurrence-free survival of 54.2 and 38.9%, respectively (*P* = 0.934 and *P* = 0.550, respectively)

The median survival time of all included patients was 32.9 months, with a median follow-up period of 21.3 months; the 3-, and 5-year OS rates were 45.3, and 32.0%, respectively. Figure 2 shows the Kaplan-Meier survival curves (a: overall survival, b: recurrence free survival) stratified by perioperative CA19–9 levels. Patients in the Responder group had a more favorable prognosis than patients in the Non-responder group (3-year OS and recurrence free survival: 50.6 and 39.9%, respectively for the Responder group and 41.6 and 20.2%, respectively for the Non-Responder group; *P* = 0.026 and *P* = 0.0621, respectively). Patients in the Responder group received a prognosis comparable to patients in the Normal group with a 3-year OS and recurrence-free survival of 54.2 and 38.9%, respectively (*P* = 0.934 and *P* = 0.550, respectively).

**Fig. 3:**
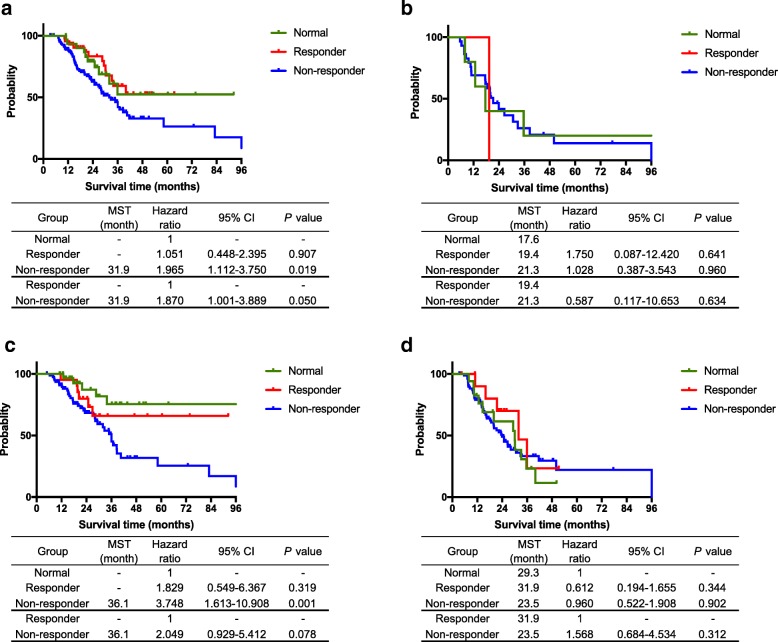
Analysis of survival time in subgroups stratified by R status and the resectability by National Comprehensive Cancer Network guideline. Among patients who underwent R0 resections, **a** the Responder group showed a significantly better prognosis than the Non-responder group (3-year OS: 52.4 and 45.7%, *P* = 0.050), and an equivalent prognosis to the Normal group (59.3%, *P* = 0.907). On the other hand, among patients who underwent R1 resections (**b**) no significant differences were observed between the Responder and Normal or the Responder and Non-responder groups (*P* = 0.641 or *P* = 0.634). Among patients with resectable pancreatic adenocarcinoma (**c**) the Responder group showed a better prognosis, with a 3-year OS of 65.9%, than the Non-responder group, with a 3-year OS of 51.1% (*P* = 0.078), and a comparable value to the 3-year OS of the Normal group of 75.5% (*P* = 0.319). On the other hand, among patients with borderline pancreatic cancer (**d**), no significant differences were observed between the Responder and Normal or the Responder and Non-responder groups (*P* = 0.344 and *P* = 0.312, respectively)

Among patients who underwent R0 resections (Fig. 3a), the Responder group exhibited a significantly better prognosis than the Non-responder group (3-year OS: 52.4 and 45.7%, respectively, *P* = 0.050), and an equivalent prognosis to the Normal group (59.3%, *P* = 0.907). On the other hand, among patients who received R1 resections (Fig. 3b), significant differences were not observed between the Responder and Normal or the Responder and Non-responder groups (*P* = 0.641 and *P* = 0.634).

Among patients with resectable pancreatic cancer (Fig. 3c), the Responder group exhibited a better prognosis than the Non-responder group (3-year OS: 65.9 and 51.1%, *P* = 0.078), and a comparable prognosis to the Normal group with 75.5% (*P* = 0.319). On the other hand, among patients with borderline pancreatic cancer (Fig. 3d), no significant differences were observed between the Responder and Normal or the Responder and Non-responder groups (*P* = 0.344 and *P* = 0.312, respectively).

### The pre-neoadjuvant therapy CA19–9 cut-off level for normalization after neoadjuvant therapy

**Fig. 4:**
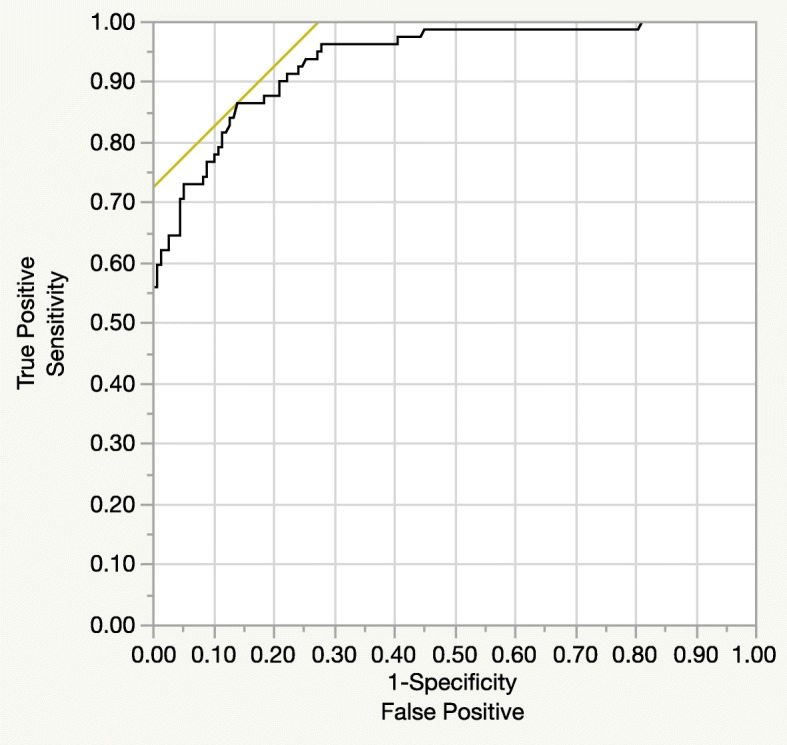
Analysis of the receiver operating characteristic curves for normalization of carbohydrate antigen 19–9 (CA19–9) levels after neoadjuvant therapy. According to this analysis, 103 U/ml was the optimal pre-neoadjuvant therapy (pre-neoadjuvant therapy) carbohydrate antigen 19–9 (CA19–9) cut-off level, defined as no elevation after neoadjuvant therapy. The area under the curve was 0.937, and the sensitivity and specificity were 86.6 and 86.1%, respectively. According to these cut-off levels, 71 of 93 patients (76%) with pre-neoadjuvant therapy CA19–9 levels < 103 U/ml did not exhibit elevated CA19–9 levels after neoadjuvant therapy

An analysis of the receiver operating characteristic curve revealed that 103 U/ml was the optimal pre-neoadjuvant therapy CA19–9 cut-off level, defined as no elevation after neoadjuvant therapy. The area under the curve was 0.937, and the sensitivity and specificity were 86.6 and 86.1%, respectively (Fig. 4). According to these cut-off levels, 71 of 93 patients (76%) with pre-neoadjuvant therapy CA19–9 levels < 103 U/ml did not display elevated CA19–9 levels after neoadjuvant therapy.

### Uni- and multivariate analyses using the Cox proportional Hazard model

Table 2 shows the results of univariate analysis of the clinicopathological factors influencing OS in the entire cohort. Pre-neoadjuvant therapy CA19–9 levels > 103 U/ml (*P* = 0.001), a pre-neoadjuvant therapy tumor size > 27 mm (*P* = 0.015), borderline resectable pancreatic cancer (*P* < 0.001), progressive disease according to the RECIST evaluation (*P* = 0.023), lymph node metastasis (*P* < 0.001), and R1 status (*P* = 0.002) were significantly correlated with a decrease in OS. According to the multivariate analysis, the pre-neoadjuvant therapy CA19–9 level (*P* = 0.010, hazard ratio: 1.711 (95% confidence interval: 1.133–2.639)), pre-neoadjuvant therapy tumor size (*P* = 0.040, 1.517 (1.018–2.278)), lymph node metastasis (*P* = 0.002, 1.905 (1.276–2.875)), and R status (*P* = 0.045; 1.659 (1.012–2.627)) were significant independent factors associated with a shorter OS (Table 2).

**Table 2:** Uni- and multivariate analysis of predictive factors for overall survival in patients undergoing surgical resection after neoadjuvant therapy

	Number	Median survival (95% CI)	*p* value by log-rank test	*p* value by Cox proportional hazards	Hazard ratio (95% CI)
Gender					
Male	119	32.7 (27.3–58.3)			
Female	121	35.7 (26.9–40.6)	0.821		
Pre-neoadjuvant therapy CA19–9 value (U/ml)					
> 103 U/ml	147	28 (23.5–32.9)			1.711
≤ 103 U/ml	93	40 (33.2-)	0.001	0.010	(1.133–2.639)
Pre-neoadjuvant therapy tumor size (mm)					
> 27	116	29.3 (23.3–34.9)			1.517
≤ 27	124	36.1 (30.1–115.7)	0.015	0.040	(1.018–2.278)
Location					
Ph	160	32.9 (26.7–36.9)			
Pbt	80	34.9 (27.3-)	0.325		
Neoadjuvant therapy					
Systematic chemotherapy	115	35.7 (30.7–58.3)			
Chemoradiotherapy	125	30.2 (27.3–40.6)	0.550		
NCCN Resectability					
Resectable	132	38.7 (33.9–99.2)			1.471
Borderline	108	24.8 (19.9–30.7)	< 0.0001	0.063	(0.979–2.219)
RESICT					
Other than progressive disease	236	33.2 (29.3–38.7)			3.930
Progressive disease	4	17.6 (12.8–18.7)	0.023	0.064	(0.912–11.740)
Duration of neoadjuvant therapy (day)					
> 69	109	35.7 (28.3–50)			
≤ 69	131	32.7 (24.9–38.7)	0.413		
Operative procedure					
Pancreaticoduodenectomy	160	32.9 (26.7–36.9)			
Distal pancreatectomy	80	34.9 (27.3-)	0.325		
N classification					
1	130	23.9 (20.4–32.9)			1.905
0	110	40 (32.7–99.2)	< 0.0001	0.002	(1.276–2.875)
R status					
1	35	19.9 (17.5–30.7)			1.659
0	205	35.9 (30.1–41.7)	0.002	0.045	(1.012–2.627)
Adjuvant therapy					
yes	205	32.9 (29.3–38.7)			
no	35	28.6 (15.2–83.1)	0.360		

NCCN National Comprehensive Cancer Network, RECIST response evaluation criteria in solid tumours

### Patterns of initial recurrence

**Fig. 5:**
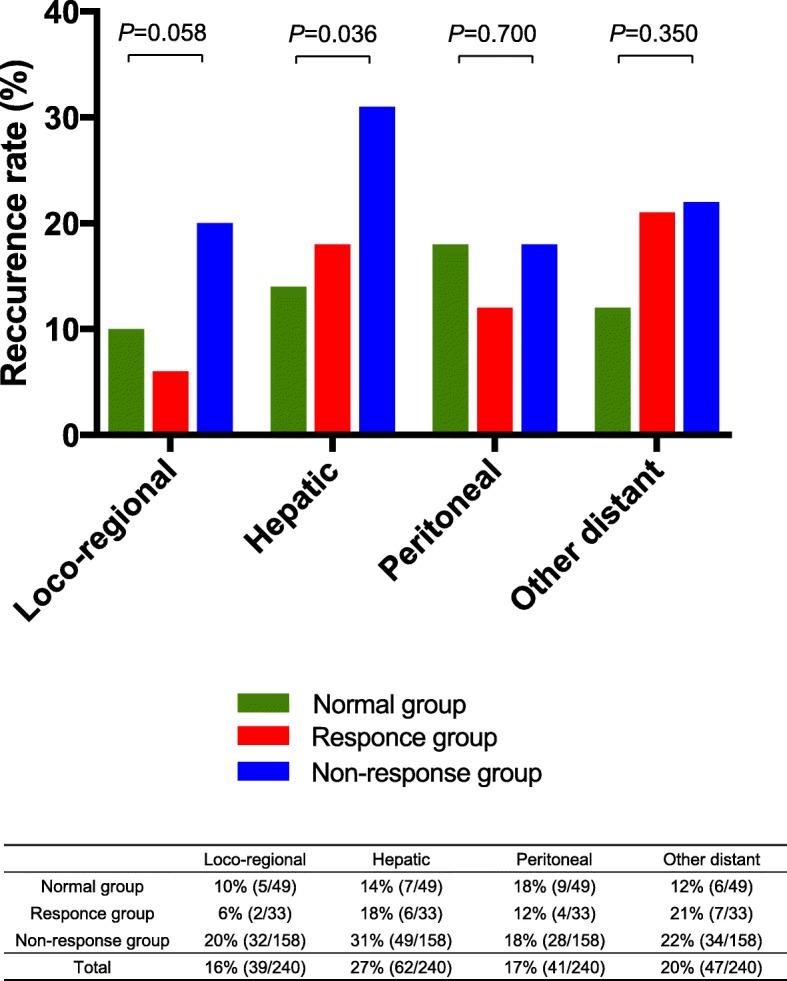
Patterns of Initial Recurrence. Initial hepatic recurrence was the most commonly observed recurrence pattern (27%), followed by peritoneal (17%), loco-regional (16%), and other distant recurrences (20%) in the entire cohort. The Normal and Responder groups showed a lower risk of hepatic recurrence (14 and 18%) than the Non-responder group (31%) (*P =* 0.036). Regarding loco-regional, peritoneal, and other distant recurrences, no significant differences were observed among the Responder and Non-responder groups (*P* = 0.058, 0.700, and 0.350, respectively)

Hepatic recurrence was the most commonly observed recurrence pattern (27%), followed by peritoneal (17%), loco-regional (16%), and other distant recurrences (20%) in the entire cohort (Fig. 5). The Normal and Responder groups showed a lower risk of initial hepatic recurrence (14 and 18%) compared with the Non-responder group (31%) (*P =* 0.036). Regarding loco-regional, peritoneal and other distant recurrences, no significant differences were observed among the three groups (*P* = 0.058, 0.700 and 0.350, respectively).

## Discussion

Despite advances in postoperative adjuvant chemotherapy, the improvement in the survival of patients with pancreatic cancer is usually modest [4–6]. A preoperative treatment combined with subsequent resection is widely used for other types of cancers, including breast and esophageal cancers [26, 27]. The purpose of this new approach for pancreatic cancer was to decrease the tumor burden and facilitate the early treatment of any micrometastatic disease [9–16]. However, the clinical profile of patients who would most benefit from neoadjuvant therapy remains unclear. In the current study, we evaluated survival and recurrence patterns by stratifying patients according to preoperative CA19–9 levels (reported to be useful surrogate markers of prognosis [17–25]), and clearly revealed a more favorable prognosis with lower incidence of hepatic recurrence for patients whose CA19–9 levels normalized during neoadjuvant therapy.

Several reports have documented the significance of CA19–9 levels during preoperative therapy [28, 29]. According to one study, alterations in CA19–9 levels during preoperative chemoradiation therapy are useful for identifying undetectable micrometastatic lesions that could potentially become distant metastases immediately after resection. However, the study was a single-institutional study with a small sample size (*n* = 64) and was based on relative stratification according to the decrease in CA19–9 levels. On the other hand, many previous papers have clarified that decreased CA19–9 levels (≤37 U/ml) measured after surgery [17–21], nodal metastasis, and R status [30–32] are independent predictors of OS in patients with no exposure to neoadjuvant therapy. In the current study, majority of patients with normal levels after neoadjuvant therapy (Normal and Responder groups) exhibited normal CA19–9 levels after surgery (78 of 82, 95%), compared with patients in the Non-responder group (86/158, 66%, *P* < 0.0001). The normalization of CA19–9 levels after neoadjuvant therapy implies a favorable response to neoadjuvant therapy treatments and predicts a better prognosis after surgery.

Elevated CA19–9 levels before surgery were considered to be an independent predictor of higher incidences of lymph node metastasis and earlier postoperative recurrence, even if R0 surgical resection was achieved [22, 23]. These elevated levels reflect systematic expansion and the existence of micrometastases in distant lesions, particularly hepatic lesions [20]. In the current study, patients in the Non-responder group showed significantly higher incidences of postoperative liver metastasis. For patients in the Non-responder group, the administration of a more powerful regimen (such as FOLFIRINOX or gemcitabine plus nab-paclitaxel) as part of neoadjuvant therapy combined with surgery, may be needed to reduce the postoperative recurrence rate.

The median duration of neoadjuvant therapy was approximately 69 days in the current study, and was determined based on the pancreatic cancer-specific neoadjuvant therapy protocols of the participating institutions. In patients showing a remarkable decrease in CA19–9 levels during neoadjuvant therapy, even if the absolute levels remained high (> 37 U/ml), several additional courses of neoadjuvant therapy might achieve normalization of CA19–9 levels before surgery, thereby resulting in a higher survival rate. Regarding locally advanced and initially unresectable pancreatic cancer, a new strategy named “adjuvant surgery” has resulted in a significantly improved OS for a highly selective patient population that responds favorably to multimodal treatment for a certain period [33–35]. Suitable candidates for “adjuvant surgery” receive nonsurgical anti-cancer treatment for more than 240 days, maintain CA19–9 levels within the lower range, and show no local progression or occult distant metastasis after various treatment modalities or surgical exploration. Regarding the administration of neoadjuvant therapy to patients with resectable pancreatic cancer, normalization of CA19–9 levels during neoadjuvant therapy might indicate optimal resection timing. In particular, for patients who exhibit a favorable response but whose CA19–9 levels are still above the normal range, several additional courses of neoadjuvant therapy may increase the possibility of CA19–9 normalization, potentially resulting in lower hepatic recurrence and longer survival after surgery. The alteration in CA19–9 levels during neoadjuvant therapy might provide important implications for preoperative treatment sequencing and patient selection for surgery.

Patients who receive neoadjuvant therapy followed by resection experience a potential survival benefit compared with patients who are initially treated with resection, particularly patients with early-stage pancreatic cancer [15]. In our study, the alteration in CA19–9 levels during neoadjuvant therapy accurately reflected the prognosis of patients with resectable pancreatic cancer. However, among patients with borderline resectable pancreatic cancer, a survival benefit wasn’t observed in the Responder group. Based on these results, an additional biomarker or combined radiological evaluation during neoadjuvant therapy might be needed for patients with borderline resectable pancreatic cancer to determine the optimal timing for resection. Otherwise, alternative presurgical treatment options (neoadjuvant therapy combined with radiotherapy, or other more powerful regimens) must be evaluated. Similar to patients with borderline resectable pancreatic cancer, altered CA19–9 levels did not predict survival in patients who underwent R1 resection. R0 resection was an independent predictor of postoperative survival.

After considering our results, we must caution the reader that we do not propose that a sustained elevation in CA19–9 levels during neoadjuvant therapy alone should be regarded as a contraindication to surgery. This study had several limitations. This study was based on retrospectively collected data from multiple institutions. Therefore, these data include heterogeneity in patient selection for administration of neoadjuvant therapy and the selection of chemotherapeutic agents. This study also excluded the patients with a total bilirubin level > 2.0 mg/dL at the time of the CA19–9 assessment, because serum CA19–9 levels may be influenced by high level of bilirubin value. However, this potentially excludes the patients with more advanced disease, in particular, who might require preoperative drainage by stenting. Moreover, this study did not include data from patients who were excluded because of metastatic or unresectable locally advanced disease observed upon restaging during the neoadjuvant therapy period or during abdominal exploration. A prospective trial with an adequate number of patients is required to accurately determine the prognostic value of decreased CA19–9 levels during neoadjuvant therapy, a prospective trial with an adequate number of patients is required.

In conclusion, normalized CA19–9 values after neoadjuvant therapy is considered to be therapeutic indicator for the effects against hepatic micrometastases. The alteration of CA19–9 values during neoadjuvant therapy indicates the optical resecting timing for subsequent surgery and the prediction of the patient prognosis after surgery.

## Conclusions

Decreased CA19–9 levels during neoadjuvant therapy predicts a better prognosis with a low incidence of hepatic recurrence after surgery.

## Additional file

Additional file 1: **Table S1.** Descriptive statistics for total cohort and three subgroups stratified by each institution. **Table S2.** Descriptive statistics for total cohort and three subgroups stratified by neoadjuvant therapy type. (PDF 55 kb)

## Abbreviations

CA19–9: Carbohydrate antigen 19–9
OS: Overall survival
RECIST: Response Evaluation Criteria in Solid Tumors
