# Supplementary material for: Decreased serum carbohydrate antigen 19–9 levels after neoadjuvant therapy predict a better prognosis for patients with pancreatic adenocarcinoma: a multicenter case-control study of 240 patients
Source: BMC Cancer. 2019 Mar 21;19:252. doi: 10.1186/s12885-019-5460-4 (PMC6427838; doi:10.1186/s12885-019-5460-4)
Supplement: Supplementary file 1 — Table S1. Descriptive statistics for total cohort and three subgroups stratified by each institution. Table S2. Descriptive statistics for total cohort and three subgroups stratified by neoadjuvant therapy type. (PDF 55 kb) [file 12885_2019_5460_MOESM1_ESM.pdf]

Supplemental table 1. Descriptive statistics for total cohort and three subgroups stratified by each institution

-	Total cohort	Subgroups		
		Normal	Responder	Non-responder
Tohoku Univ., n (%)	71	12 (17)	4 (6)	55 (77)
Nara Univ., n (%)	56	10 (18)	12 (21)	34 (61)
Kansai Univ., n (%)	55	12 (22)	8 (15)	35 (63)
Komagome, n (%)	24	7 (29)	4 (17)	13 (54)
Hiroshima Univ., n (%)	17	4 (23)	3 (18)	10 (59)
Wakayama Univ., n (%)	15	3 (20)	2 (13)	10 (67)
Kobe Univ., n (%)	2	1 (50)	0 (0)	1 (50)
Total, n (%)	240	49 (20)	33 (14)	158 (66)

Supplemental table 2. Descriptive statistics for total cohort and three subgroups stratified by neoadjuvant therapy type

-	Total cohort	Subgroups			P value
		Normal	Responder	Non-responder	
**Chemotherapy**					
Gemcitabine	23 (10)	3 (13)	3 (13)	17 (74)	0.734
Gemcitabine + S-1	91 (38)	23 (25)	8 (9)	60 (66)	
Other regimens	1 (0)	1 (100)	0 (0)	0 (0)	
Total, n (%)	115 (48)	27 (23)	11 (10)	77 (67)	
**Chemoradiotherapy**					
Gemcitabine	64 (27)	10 (16)	14 (22)	40 (62)	0.018
S-1	49 (20)	9 (18)	7 (14)	33 (68)	
Gemcitabine + S-1	10 (4)	3 (30)	1 (10)	6 (60)	
Other regimens	2 (1)	0 (0)	0 (0)	2 (100)	
Total, n (%)	125 (52)	22 (18)	22 (18)	81 (64)	
